# Impact of sanitation system types on residential and environmental presence of human waste and parasites in Alabama

**DOI:** 10.1186/s40249-025-01334-4

**Published:** 2025-07-11

**Authors:** Brandon Hunter, Catherine Coleman Flowers, Rojelio Mejia, Marc Arnold Deshusses

**Affiliations:** 1 Department of Civil & Environmental Engineering, Duke University, Durham, NC USA; 2 Center for Rural Enterprise & Environmental Justice, Madison, AL USA; 3 Center for Diverse Leadership in Science, UCLA, Los Angeles, CA USA; 4 Nicholas School of the Environment, Duke University, Durham, NC USA; 5 Department of Pediatrics, Tropical Medicine, Baylor College of Medicine, Houston, TX USA; 6 Natural Resources Defense Council, Washington, DC USA

**Keywords:** Sanitation, Infrastructure, Waste, Parasite, Exposure

## Abstract

Lowndes County is a predominantly Black rural county in Alabama, in the United States, which has a historical and current legacy of racial discrimination, creating inequitable infrastructure access and adverse health impacts. Over 80% rely on on-site sanitation infrastructure and most are failing. A community assessment of exposure to untreated sewage was conducted using samples from residential drinking water, surface swabs, and soil combined with environmental water and soil samples using culture-based and quantitative polymerase chain reaction (qPCR) methods. Testing varied slightly across samples, due to difficulty of access or availability. Of 43 households, 68% and 55% of houses had detectable presence of human fecal matter indicator in their soils and on their doorsteps, respectively, and 0% had detectable amounts of culturable *Escherichia coli* in their drinking water. Of 40 houses sampled, 88% tested positive for *E. coli* in soil samples. Of 39 residences, 31% had positive presence of environmental and zoonotic parasites in soil, but none for *Necator americanus*, *Cryptosporidium* species*,* or *Giardia intestinalis*. Of the 18 sampled environmental surface waters, 100% tested positive for culturable *E. coli*, 50% had detectable human fecal matter indicator present, and 27% tested positive for anthropogenic parasites. This work sheds light that there is presence of culturable *E. coli,* human fecal matter, and anthropogenic parasites in residential soil samples of all sanitation types (municipal, septic tank, and straight piping) and in environmental surface waters throughout the sampled areas. Our findings support the narrative that sanitation infrastructure of all types in Lowndes County, Alabama are compromised and highlights residential and environmental exposure to raw wastewater.

**Graphical Abstract:**

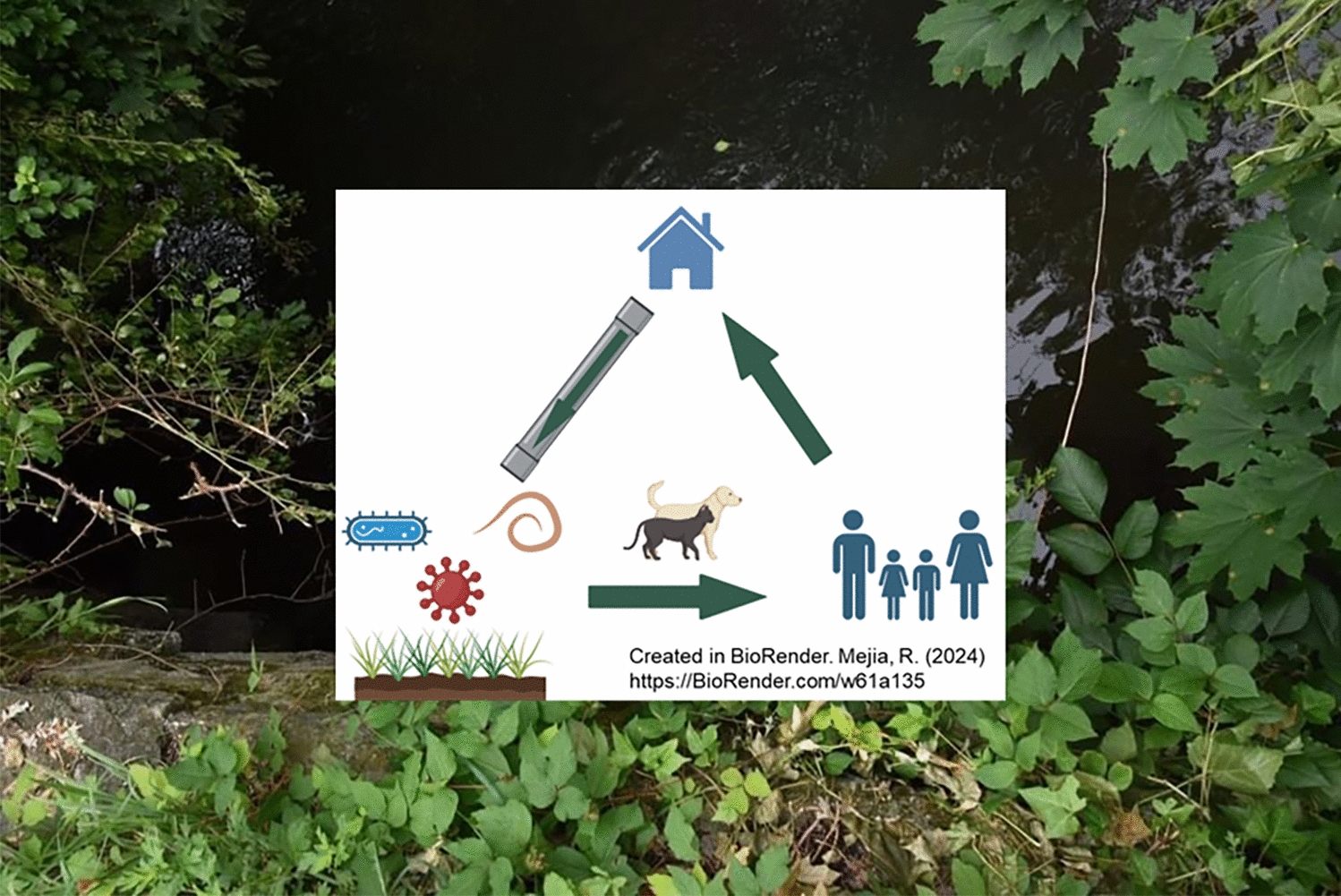

**Supplementary Information:**

The online version contains supplementary material available at 10.1186/s40249-025-01334-4.

## Background

In 2022, the World Health Organization estimated that 3.4 billion people (almost two-thirds of the global population) lacked access to safely managed sanitation (hygienic management of human waste and wastewater), of which about 900 million people practiced open defecation [1]. While inadequate sanitation infrastructure is predominately found in low-income nations, middle- and high-income countries also suffer. For example, in the United States, with many wastewater systems being over a century old, deteriorating, and failing, over 1.7 million people lack access to adequate plumbing and the country has a D + rating for sanitation infrastructure quality [2–7]. Failing sanitation infrastructure affects some groups disproportionately, often with the most marginalized communities and demographics being most negatively impacted [8]. For example, race and its basis of discrimination is the strongest predictor of which communities have access to safe and affordable sanitation [9, 10].

The “Black Belt” region of the American South, named for both its rich black topsoil for plantation crops and for its racial demographics, has deep historic and currently legacies of institutionalized discrimination, racialized violence, and economic oppression, all of which still manifest in many forms, including inequitable access to safe and affordable sanitation infrastructure. About 74% of Black Belt residents are Black, 32% live in poverty, and 98% who are below the poverty line are Black. About 82% of residents rely on septic tanks and live in rural areas, about 90% of on-site sanitation systems in the Black Belt are poorly functioning or failing (not effectively containing, transporting, and/or treating waste), and over 40% of residents report raw sewage backup into their homes [11, 12].

A study from Baylor College of Medicine found that in a small community in Lowndes County, Alabama which has improper septic infrastructure, of those who were evaluated, over 34% of adults tested positive for *Necator americanus*, a parasitic helminth otherwise known as hookworm (formerly thought to be eradicated in the U.S.) [12]. In that same study other Lowndes County residents tested positive for *Strongyloides stercoralis*, *Toxocara*, *Entamoeba histolytica*, and *Cryptosporidium* – hookworm (2013 study) [12]. These results contrast several other studies including a more recent large-scale study using molecular diagnostics which did not detect these intestinal parasites in Alabama [13]. Both studies reported that exposure to raw sewage is a significant problem. This phenomenon may very well worsen as the effects of climate change add stress to the already aging sanitation infrastructure. With increasing numbers of floods, droughts, and extreme weather events, aging and vulnerable sanitation infrastructures are experiencing more system stresses. Increased rainfall in Alabama areas, can result in floods and rising underground water tables, which increase the hydraulic load on sanitation systems and increases the frequency and magnitude of infrastructure failure, leading to more health issues [14–16].

The goal of this study seeks to determine if there is evidence of untreated sewage in and around people’s homes or evidence of untreated sewage in the natural environment. We investigated the prevalence of exposure to fecal contaminants in Lowndes County, Alabama by assessing presence and magnitude of fecal indicators, fecal bacteria, and anthropogenic and animal parasites in residential and environmental contexts. To determine if samples contained fecal bacteria that are harmful to humans, the study performed quantitative assessment of culturable *Escherichia coli.* To determine if samples included untreated human waste a specific DNA marker that is exclusive to humans and only found in human fecal matter, called HF183, was tested for. Both cultural bacteria tests (*E. coli*) and DNA marker tests for sewage (HF183) were tested for all sample types. Residential drinking water samples were collected to evaluate the quality of water people consume. Residential soil samples and swab samples of residential doorsteps were collected to evaluate prevalence of human waste in yards and if human waste could be potentially tracked inside or outside of dwellings. Environmental samples were collected to determine the extent to which human waste exists beyond theoretical containment of residential sanitation infrastructure.

## Methods

### Participatory consent

Those who participated in the residential collection of drinking water, soil on the residential property, and swab sampling of residential doormats agreed to be involved in the study through grassroots efforts led by the Center for Rural Enterprise & Environmental Justice (CREEJ). CREEJ community surveyors directly asked people to participate in this study, mostly based on relationships which were developed through decades of combinations of previous research efforts, community meetings, community initiatives, general outreach efforts, and helping community members access resources for sanitation issues and rural development issues. We designed our study to also be flexible and accommodating for interested Lowndes County residents who might have developed interest by learning about the study through word-of-mouth over the course of the sample collection period. All participating residents provided their consent to participate through a Duke University IRB approved protocol (IRB #2017-1391) in July 2019.

### Sanitation system classification

We segmented the participating residences into three different categories, based on what type of sanitation infrastructure the dwelling used – municipal systems, septic tank systems, or straight piping systems. Municipal sanitation systems are those in which wastewater from a large-scale pipeline network, which interconnects many different wastewater-generating locations, is transported to and treated at a centralized location, typically a wastewater treatment facility. Municipal sanitation systems typically have complete primary treatment, complete secondary treatment, and tertiary treatment, depending on the use case of the final effluent (output water). Typically, municipal utilities are responsible for collection, transportation, and treatment of sewage and are paid by individual wastewater-generating point sources for the services. Septic tank sanitation systems are generally used in a smaller-scale cluttered context that connects a few wastewater-generating sources or it is used in a single-use context where one wastewater-generating source has its own septic tank ‘on-site’. In this study, all residential septic tanks were single-dwelling contexts. Septic tank systems typically have partial primary treatment and partial secondary treatment. Typically, dwelling residents are responsible for the maintenance of the sanitation system. Straight piping sanitation systems are generally used at the individual building context in which there is no primary, secondary, or tertiary treatment at all. Typically, wastewater from straight piping systems is deposited on top of the ground on the same site that the wastewater is generated from. We designed our study to have samples from all three categories of sanitation infrastructure types.

### Sample collection and testing

For assessment of residential contexts, soil samples were collected on the property grounds of the participants. Drinking water samples and swabs samples of inside and outside residential front doorstep surfaces were collected from residential locations to evaluate potential for exposure to untreated human waste. Soil and swab samples were used to explore their possible relationship with different sanitation infrastructure types. To evaluate possible fecal exposure in environmental contexts, soil and water samples from environmental surface water bodies were collected to determine if environmental samples were contaminated with human fecal matter.

### Residential drinking water sample collection

Drinking water samples were collected from each participating resident from their kitchen faucet. To minimize biological contamination of the samples, faucet ends were cleaned with a 70% ethanol solution. After allowing the kitchen tap to run for 1 min, approximately 2 L of water was collected in 4 mm thick, sterile plastic bags (VWR International, LLC, USA). Residential drinking water samples in bags were immediately placed on ice until returning to the lab and stored at 4 °C until processed. All drinking water samples were processed within 6 h of collection. The collection of sterile, autoclaved water was used for a negative control as both a field blank and experimental blank.

### Residential soil sample collection

Residential soil samples were collected from each participating resident from within the property boundaries that the resident lived on. Because of the diversity of sanitation systems present for each resident dwelling, various methods were implemented for soil collection. All soil samples were collected within the top 2-inch layer of soil with sterile 50 ml centrifuge tubes. When samples of interest had high moisture content, a sterile wooden applicator stick was used to transfer soil into tubes. For residences with septic tanks, soil samples were collected from areas within 0.5 m adjacent to waste pipes coming out of the home structure, in areas within 0.5 m adjacent to the septic tank, and low-lying areas of their yards. For residences which were connected to a municipal sewage system, soil samples were collected 0.5 m away from waste pipes out of the home and in low-lying areas. For residences that had “straight-piping”, soil samples were collected 0.5 m from the opening of the straight pipe and in low-lying areas. Soil samples were stored on ice until processed or stored in the lab. All soil samples were processed within 6 h of collection. Processing included conducting culture-based IDEXX Colilert Quanti-tray 2000 (Westbrook, Maine, USA) tests, followed by freezing at − 20 °C for molecular biology analysis.

### Residential swabbing sample collection

Surface swabs were collected on the floor surface directly inside and directly outside of the door that participants used most frequently. A sterile silicon template of 342 cm^2^ (38 cm × 9 cm) was placed on the indoor and outdoor floor surfaces, in the center of the door frame, 10 cm away from the door inside and outside. The template was placed on top of whatever surface was found inside or outside of the door (e.g., wood floor, carpet, wood step, concrete step, brick step, welcome mat, etc.). Sterile HydraFlock swabs (Puritan, Guilford, ME) were dipped into a sterile phosphate buffered solution and swabbed across the entire surface within the template boundary. Swab samples were used with a firm stroke over two passes, perpendicular to each other. One swab was used for each separate (indoor and outdoor) doorstep sample collections. Swabs were rotated during stoke passes and collected in duplicate. Swab tips were aseptically transferred into sterile 15 ml centrifuge tubes. Swab samples were placed on ice until returning to the lab and were processed within 6 h of collection. Processing included conducting the IDEXX Colilert Quanti-tray 2000 (Westbrook, Maine, USA) culturable *E. coli* tests, followed by freezing at − 20 °C for later quantitative polymerase chain reaction (qPCR) molecular biology analysis.

### Environmental surface water and soil sample collection

Surface water samples of 2 L volume were gathered from ponds and streams that were identified as points of interest by community partners. Each location comprised of two subsamples: a water subsample and a soil subsample. The water subsamples were collected with a sterile water sampling dipper, 3 m away from the water’s edge, and 6 inches below the surface of the water. For soil samples which were collected at surface water points, soil was collected within 15 cm above the surface water shore. Samples were immediately placed on ice until returned to the lab. All water samples were processed within 6 h of collection. Processing included analysis with the IDEXX tests, followed by freezing for further molecular biology analysis. Collection of sterile water into the water sampling dipper was used as a negative control. Samples from 2–8 sites were collected per field sampling day. Given that all of the samples must be processed for IDEXX tests, it was deemed logistically infeasible to have adequate time to filter all water samples with high particulates within the same day. To standardize the filtering of surface water samples, all samples were frozen within 6 h of collection to preserve DNA. Samples were then thawed, filtered through a 0.2 µm-pore-size Supor-200 filter (Pall Co., Port Washington, NY), and immediately processed for DNA extraction. After DNA extraction, samples were immediately frozen at − 20 °C.

### Coliform testing

Total coliform and *E. coli* were quantified by fluorescence of rapid, culture-based IDEXX Colilert Quanti-tray 2000 (IDEXX Laboratories, Westbrook, ME) following the manufacturer’s recommended protocol. The IDEXX pouches were sealed and then incubated at 35 °C for 24 h. An ultraviolet (UV) light was used to count total coliforms and fecal coliforms. The IDEXX MPN Generator software version 1.4.4 was used to convert colored and illuminated well counts into MPN values with a 95% confidence interval. The detection limits were 1 CFU ml^−1^. For residential drinking water and environmental surface water samples, undiluted samples were divided into 100 ml aliquots (portions) for analysis. For all residential and environmental soil samples, 5 g of wet soil was added to 95 ml of sterile deionized (DI) water and vortexed (mixed) for 1 min. An additional serial dilution was prepared and vortexed for 1 min. A 100 ml aliquot of each dilution was put into respective IDEXX trays and incubated at 35 °C for 24 h. After swabbing the surface of doormats, the swab tips with the samples were aseptically added to 100 ml of sterile DI water and vortexed for 1 min. The resulting 100 ml solution of water, IDEXX reagents, and suspended matter from the swabs were poured in its entirety into respective IDEXX testing trays.

### DNA extraction

DNA from environmental surface water, residential and environmental soil, and residential doorstep swab samples were extracted using Qiagen PowerSoil DNA extraction kits, its associated protocol (MO BIO Laboratories, 2016), and a BioSpec Mini-Beadbeater-16 (BioSpec Corp., Bartlesville, OK). Modifications were made to the recommended Qiagen protocol by heating the extraction kit solution ‘C1’ to 60 °C before adding to the DNA extraction bead tube, heating the solution ‘C6’ to 55 °C before its utilization, conducting the elution step with 30 µl in two 15 µl elution (removal) steps, and waiting 5 min after addition of the solution ‘C6’ before centrifuging (physically separating the liquid component from the solid components). Modifications for the mass of sample used and the duration of bead beating were made after conducting DNA extraction optimization assays. Soil samples were thawed, and 0.5 g was aseptically transferred to a bead beater for 3 min. Swab samples were thawed and aseptically transferred to a bead beater and processed for 3 min. Each water sample was passed through a 0.2 µm-pore-size Supor-200 filter (Pall Co., Port Washington, NY). Water samples were stirred and passed through filters until the filters were clogged. Filters were aseptically cut into fine pieces and transferred into a bead beater and processed for 5 min.

### Human source tracking methods

All samples were tested to determine if there was presence of a specific DNA marker called HF183, that is exclusive to humans and only found in human fecal matter sources. To test for presence of the HF183 gene marker in samples (source tracking), HF183/BacR287 DNA primers and the BacP234MGB DNA probe were used to quantify HF183 Bacteroides 16S rRNA, as a human-specific genetic fecal marker. To create the solution to perform the qPCR HF183/BacR287 assay, the simplex reaction 20 µl well mixtures included 10 µl of 2 X SsoAdvanced Universal Probe Supermix (Bio-Rad, Hercules, CA), 1 µmol/L of each forward and reverse DNA primer (IDT DNA, Coralville, Iowa), 80 nmol/L of 6-carboxyfluorescein (FAM)-labeled probe (Thermo Fisher Scientific, Waltham, MA), 0.2 µg/µl bovine serum albumin (Sigma-Aldrich Corp., St. Louis, MO) and microbial grade water (Qiagen, Germantown, MD). The thermocycling conditions of performing the qPCR analysis were 95 °C for 3 min, followed by 40 cycles of 95 °C for 15 s, and 60 °C for 30 s. All samples were evaluated in triplicate with respect to a HF183 template standard curve on the same 96-well plates. Limits of Detection (LOD) C_q_ (cycles of quantification) values ranged from 39.68 to 37.97 cycles and Limits of Quantification (LOQ) C_q_ values ranged from 38.49 to 35.17 cycles. The HF183 assay yielded a standard curve with r^2^ values ≥ 0.99 and DNA magnification cycle efficiencies between 90–110%. The range of quantification was from 86-227036 copies g^−1^ soil of the reference HF183 DNA template, compared to a no-template negative control in triplicate. Samples that had values between the LOD and the LOQ are reported as 1 copy g^−1^ soil.

### Parasite methods

To detect parasite DNA in soil samples, the following methods were used [16]. A total of 128 soil samples were collected using 50 ml sterile test tubes and stored in airtight plastic containers and stored on ice until processed in the laboratory. Upon arrival in the laboratory, the samples were weighed. To streamline handling, each sample was then portioned into new sterile Falcon tubes, ensuring that no more than 25 ml of solid material was placed in each tube. In cases where a sample exceeded this volume, it was evenly distributed between two tubes. A solution of 0.05% TWEEN/PBS was introduced into each tube until the total volume reached 45 ml, followed by 5 min mixing and then centrifugation at 500 × *g* for 5 min. The resulting supernatants (remaining floating liquids) were then filtered using a 3.0 µm pore-sized hydrophilic mixed cellulose esters membrane from Millipore, (Tullagreen, Ireland). These membranes underwent processing utilizing the FastDNA SPIN Kit for Soil from MP Biomedicals, (Santa Clara, California, USA). Subsequent to a heating step at 90 °C for 10 min, the DNA was subjected to analysis through multi-parallel qPCR. This analytical method aimed to screen for 11 distinct parasitic-specific DNA sequences, including *Ancylostoma duodenale, Ascaris lumbricoides, N. americanus, S. stercoralis, Toxocara canis/cati, Trichuris trichiura, Blastocystis* species*, Cryptosporidium* species*, Entamoeba histolytica, and Giardia intestinalis*, using DNA primers and probes from previous research, (Supplemental Table 1 and Supplemental Table 2) [17]. All parasite primers and probes were tested against genomic parasite DNA from each species with no cross-reactivity noted.

**Table 1:** Presence of each parameter measured of all samples

				Residential samples		Environmental samples
			Municipal	Septic tank	Straight piping	All
			(*n* = 6)	(*n* = 25)	(*n* = 12)	(*n* = 18)
Water samples	Bacterial	*Escherichia coli*	0 (*n* = 6)	0 (*n* = 25)	0 (*n* = 12)	16 (*n* = 16)
	Source tracking	HF183	–	–	–	9 (*n* = 16)
Swab samples	Bacterial	*E. coli*	3 (*n* = 6)	5 (*n* = 25)	5 (*n* = 12)	–
	Source tracking	HF183	5 (*n* = 6)	12 (*n* = 24)	6 (*n* = 12)	–
Soil samples	Bacterial	*E. coli*	6 (*n* = 6)	22 (*n* = 24)	10 (*n* = 10)	16 (*n* = 16)
	Source tracking	HF183	5 (*n* = 6)	16 (*n* = 24)	7 (*n* = 10)	4 (*n* = 16)
	Parasites	*Ancylostoma duodenale*	0 (*n* = 6)	0 (*n* = 23)	0 (*n* = 10)	0 (*n* = 15)
		*Ascaris lumbricoides*	0 (*n* = 6)	1 (*n* = 23)	0 (*n* = 10)	0 (*n* = 15)
		*Necator americanus*	0 (*n* = 6)	0 (*n* = 23)	0 (*n* = 10)	0 (*n* = 15)
		*Trichuris trichiura*	0 (*n* = 6)	1 (*n* = 23)	0 (*n* = 10)	0 (*n* = 15)
		*Strongyloides stercoralis*	0 (*n* = 6)	0 (*n* = 23)	0 (*n* = 10)	0 (*n* = 15)
		*Giardia intestinalis*	0 (*n* = 6)	0 (*n* = 23)	0 (*n* = 10)	0 (*n* = 15)
		*Blastocystis hominis*	2 (*n* = 6)	3 (*n* = 23)	4 (*n* = 10)	3 (*n* = 15)
		*Entamoeba histolytica*	1 (*n* = 6)	0 (*n* = 23)	0 (*n* = 10)	1 (*n* = 15)
		*Cryptosporidium*	0 (*n* = 6)	0 (*n* = 23)	0 (*n* = 10)	0 (*n* = 15)
		*Toxocara canis*	0 (*n* = 6)	1 (*n* = 23)	0 (*n* = 10)	0 (*n* = 15)
		*Toxocara cati*	0 (*n* = 6)	1 (*n* = 23)	0 (*n* = 10)	0 (*n* = 15)
	Bacterial, source tracking, and parasites	*E. coli*, HF183, and any parasite (excluding *Toxocara*)	3 (*n* = 6)	4 (*n* = 22)	3 (*n* = 10)	3 (*n* = 15)

### Statistical analysis methods

Non-normal variables were reported as median and interquartile ranges. Tukey Tests were used to test the hypothesis of the average concentrations of the same testing parameter (e.g. *E. coli,* HF183, and parasites) did not significantly differ when comparing and contrasting potential differences based on residences having a particular type of sanitation type (municipal, septic tank, or straight piping). Spearman Tests were conducted to test the hypothesis that there is no significant association between two testing variables, in the cases where all sanitation system types are aggregated or disaggregated. Linear regressions were performed to understand the predictive connection between the concentrations of different testing parameters, both within and outside of sanitation system type groups, testing the hypothesis that there no significant relationship between independent and dependent variables. These statistical tests were performed to test if the magnitude of concentration of testing parameters, in isolation or in combination with other parameters, influenced the magnitude of other testing parameters in the same sample. Logistic regressions were performed to test hypotheses that there is no significant relationship between the presence and absence of dependent and independent variables. These statistical tests were performed to evaluate if the presence of one or more testing parameters influenced the presence of another testing parameter, with respect to all samples collected and when evaluating samples from houses that had the same sanitation system type. These tests were chosen to determine if there is a strong relationship between fecal indicators in collected samples, and if any relationships between parameters varied based on the type of sanitation system a resident has in Lowndes County.

## Results

### Household participation

About 50 residents were invited by CREEJ community surveyors and all agreed to participate. However, some were not available when the sampling team arrived to their residences, so their samples were not collected. Others who heard about the study through word of mouth and asked the research team to volunteer to participate. Of the 43 total study participants, most of whom were originally recruited by CREEJ, 6 residents had municipal sanitation systems, 25 residents had septic tank systems, and 12 residents had straight piping system (see Table 1). All participating residents were Black. Most of the participants were considered to be in low-income contexts and some were considered to be in medium-income contexts. Most of the participants lived in rural settings and some lived in the city limits. The study participants were generally evenly distributed across all of the Lowndes County districts, excluding sampling Fort Deposit from which there was no sampling. From decades of doing work throughout the county, CREEJ is under the impression that the study sample is closely representative of the larger Lowndes County population, with respect to race, income, sanitation system context, and geographical distribution. Not all sample types were collected from each sample site, due to difficulty of access or availability of sampling materials. For example, of the total 25 houses with septic tanks that were sampled, only 24 of those had soil samples, and only 23 of those soil samples were tested for parasites due to the availability of sampling materials during field collection times. More details can be found in Table 1.

### Fecal markers & parasites in residential drinking water & soil

**Fig. 1:**
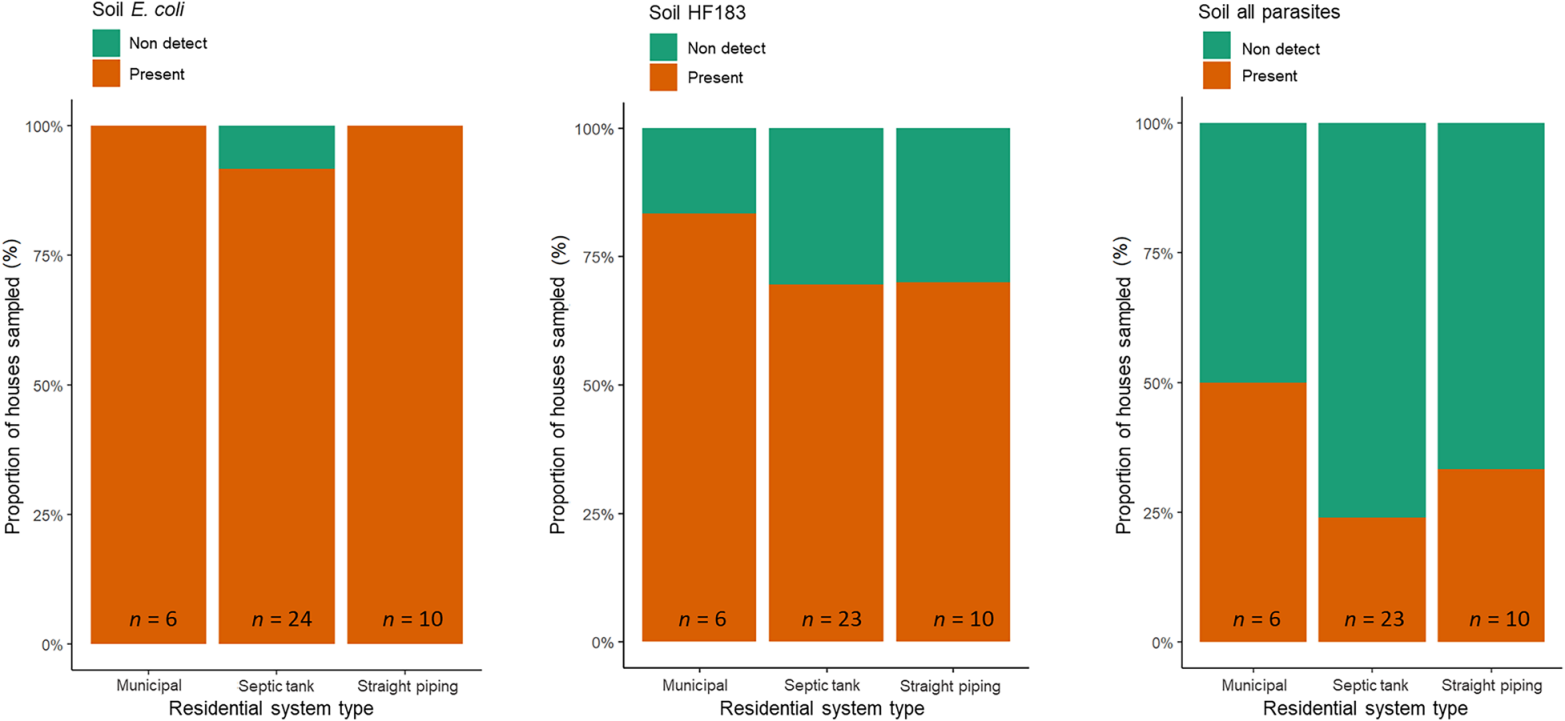
Proportion of houses which had detectable levels of *Escherichia coli*, HF183 and all parasites in residential soil samples

**Fig. 2:**
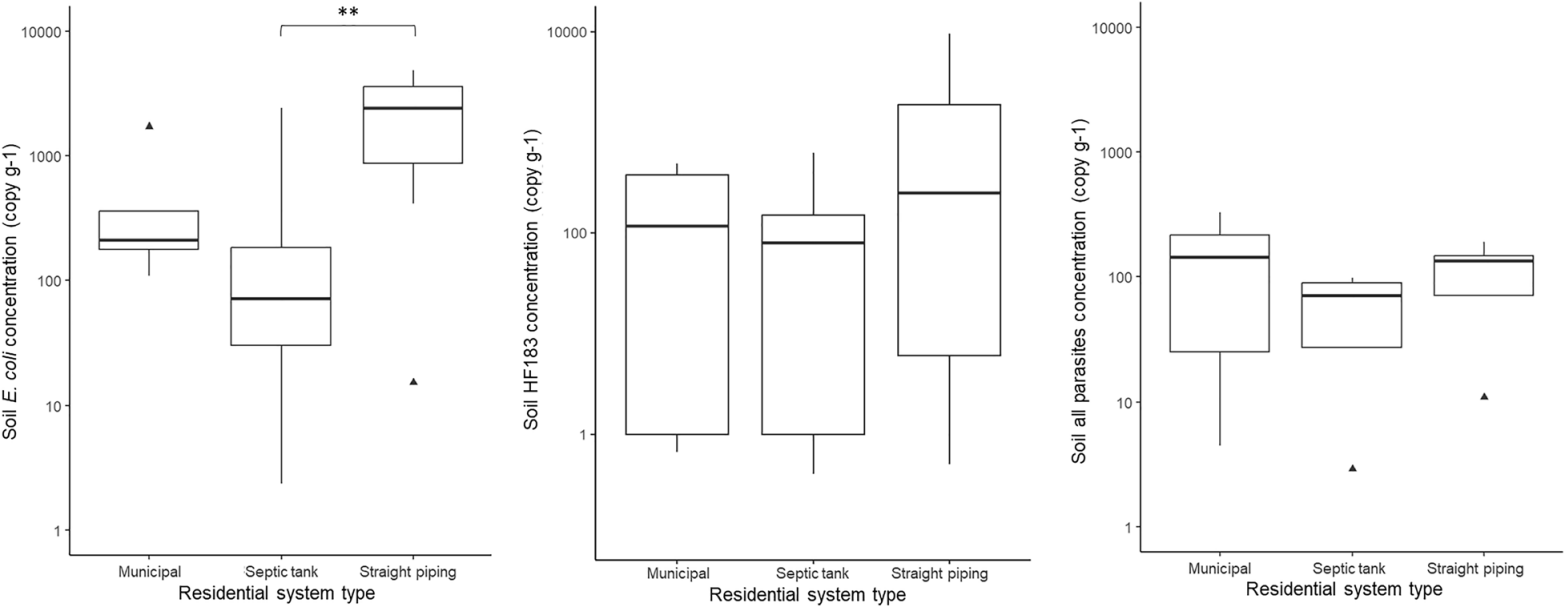
Boxplots of quantifiable average *Escherichia coli*, HF183 and all parasites concentrations in residential soil samples; Samples that had values between the LOD and the LOQ are reported as 1 copy g^−1^ soil;^▲^outliers; ^**^statistical significance (*P* < 0.01) from ANOVA test

Of the 43 residential homes drinking water samples, none had detectable (1 MPN 100 mL^−1^ detection limit) amounts of culturable *E. coli*. Of the 40 houses sampled for bacterial contamination of soil, 88% tested positive for *E. coli* in soil samples, as shown in Fig. 1. *E. coli* being present in the soil of 88% of residences indicates fecal presence is widespread. The Alabama samples ranged from non-detect to 4840 *E. coli* CFUs g^−1^ wet soil, a few samples reaching the IDEXX upper limit of detection (4840 CFUs g^−1^ wet soil) even after a ten-fold dilution. A box plot of the *E. coli* concentrations for the residential soil samples which had quantifiable amounts is shown in Fig. 2. A Tukey test was performed which showed a statistically significant difference (*P* < 0.01) between the average soil *E. coli* concentration between houses with straight piping and those with septic tank systems.

As shown in Fig. 1, the soil of 72% of 39 houses sampled contained detectable amounts of the human-specific fecal marker gene HF183, which is specific to human fecal matter and is increasingly used to identify sewage presence [19]. For houses on municipal services, septic tank, and straight piping systems, respectively, 83%, 70%, and 70% had detectable amounts of HF183 gene markers in the soil. Concentrations ranged from non-detect to 2.27 × 10^5^ copies g^−1^ wet soil and also demonstrated no statistical significance in the average among sanitation system types, as per Tukey tests (Fig. 2).

The soil adjacent to 39 houses was tested for parasites, with the following positive results: *A. lumbricoides* (1 out of 39, 3%)*, N. americanus* (0, 0%)*, A. duodenale* (0, 0%), *Trichuris trichiura* (1, 3%)*, S. stercoralis* (0, 0%)*, G. intestinalis* (0, 0%), *B. hominis* (9, 23%)*, Entamoeba histolytica* (1, 3%)*, Cryptosporidium* species (0, 0%)*, Toxocara canis* (1, 3%)*, and Toxocara cati* (1, 3%), respectively. These results indicate a relatively low incidence of parasites in the soils; detailed results are reported in Supplemental Table 3. Excluding the exclusive zoonotic *Toxocara* which not transmitted from person to person and is not directly linked to sanitation issues*,* the soil adjacent to 12 residences (31%) tested positive for at least one species of parasite (Fig. 1, Supplemental Table 3 and Supplemental Table 4). With respect to residences with different sanitation system types, prevalence was highest for houses connected to municipal sewerage (50%), followed by straight piping (40%) and then septic tanks (22%), as shown in Fig. 1. A linear regression demonstrated that for residences who had septic tank systems, there was a statistical significance (*P* < 0.05) for correlation between concentrations of HF183, *E. coli,* and *A. duodenale.*

**Fig. 3:**
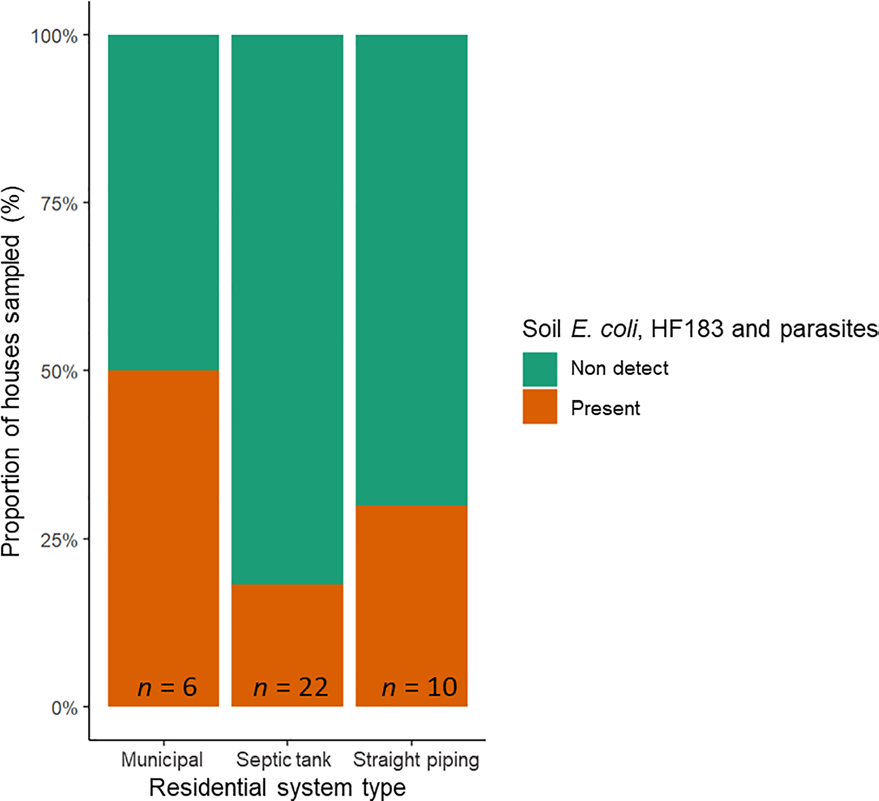
Proportion of houses which had detectable levels of *Escherichia* *coli*, HF183, and parasites all present in residential soil samples where all three contaminant types were sampled

As shown in Fig. 3, of the 38 residences which soil was tested for *E. coli*, HF183, and parasites, 10 of the houses (26%) had soil samples that tested positive for all three categories simultaneously. Residences relying on municipal, septic tank, and straight piping infrastructure had 50%, 18%, and 30%, respectively, of each group that tested positive for all three categories simultaneously.

### Fecal markers in residential swabs

**Fig. 4:**
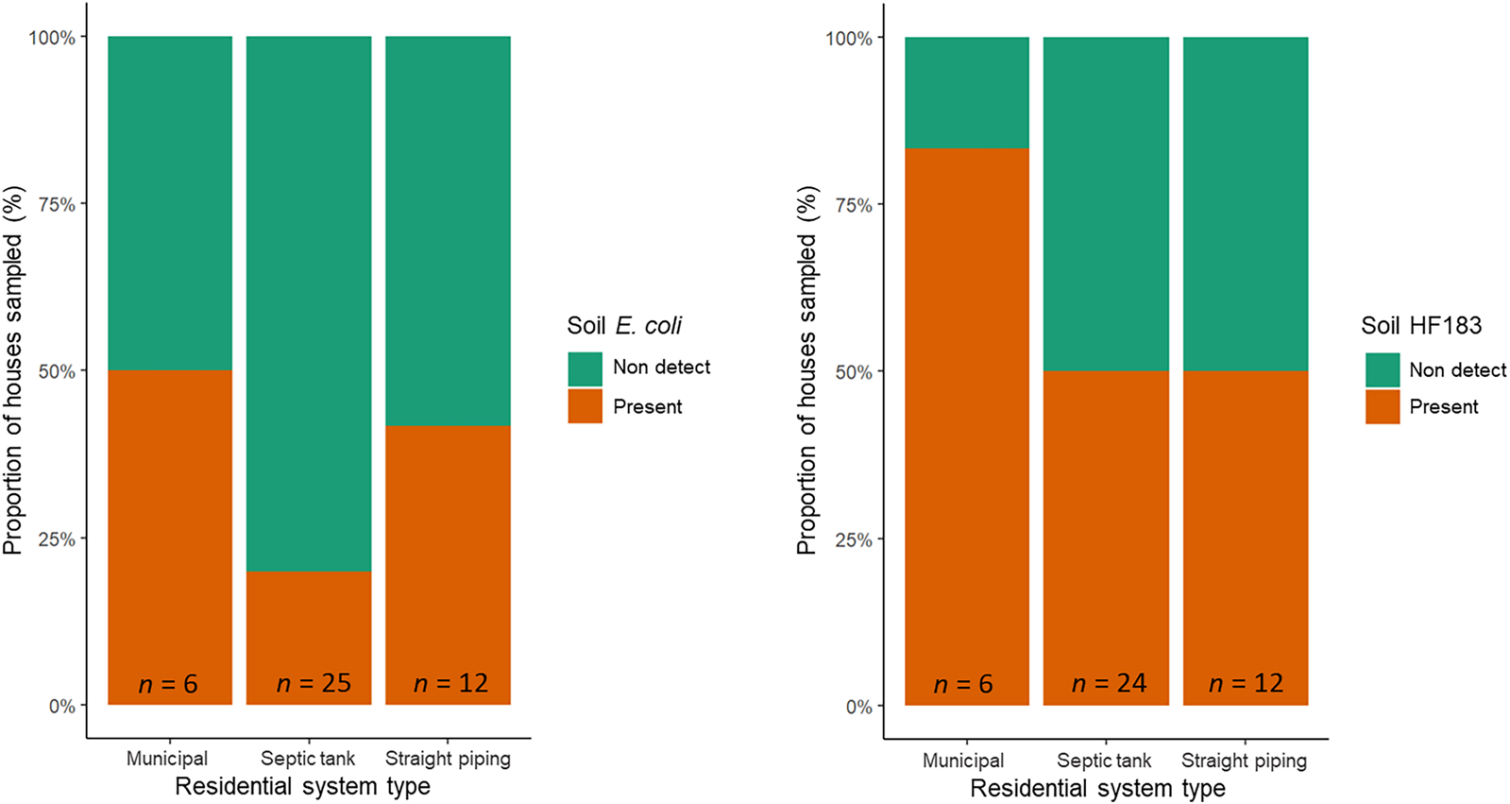
Proportion of houses which had detectable levels of *Escherichia* *coli* or HF183 in residential swab samples

**Fig. 5:**
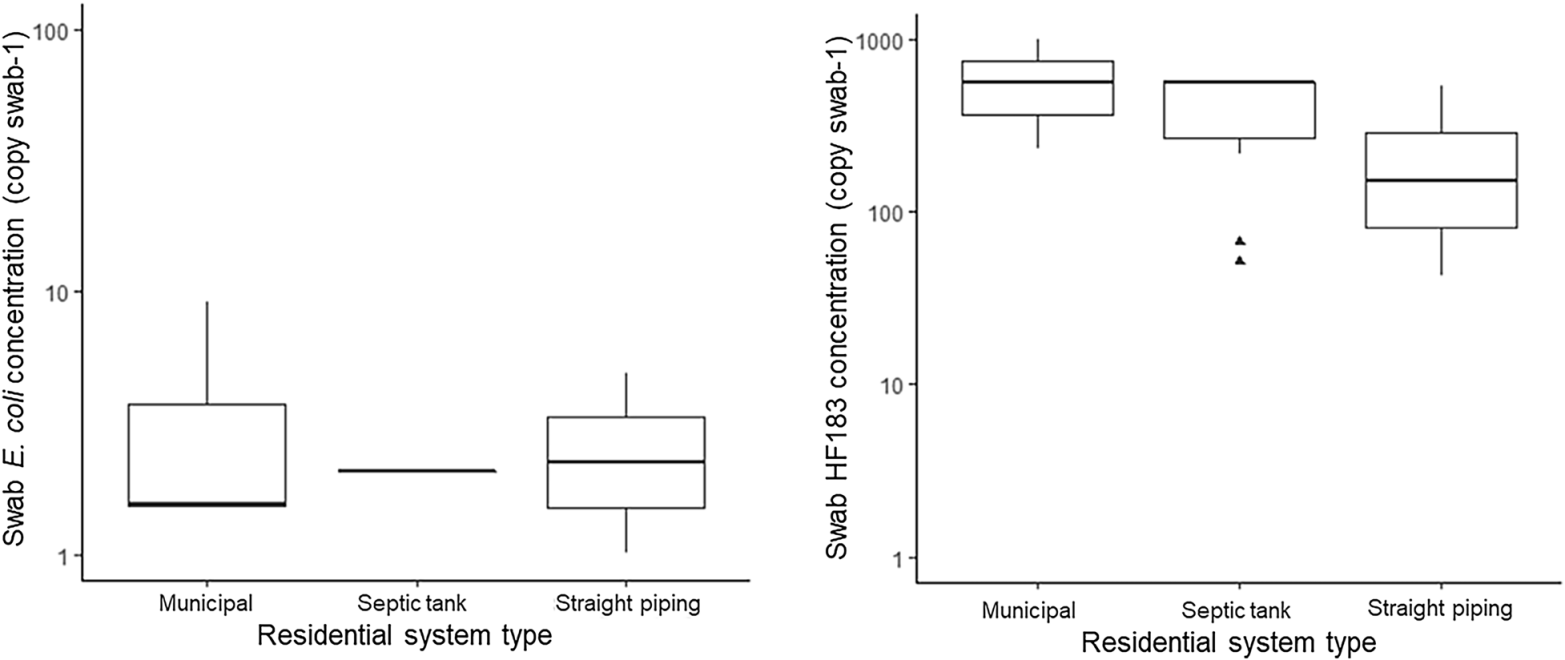
Boxplots of average quantifiable *Escherichia* *coli* or HF183 in residential swab samples. Samples that had values between the LOD and the LOQ are reported as 1 copy swab^−1^; ^▲^outliers

**Fig. 6:**
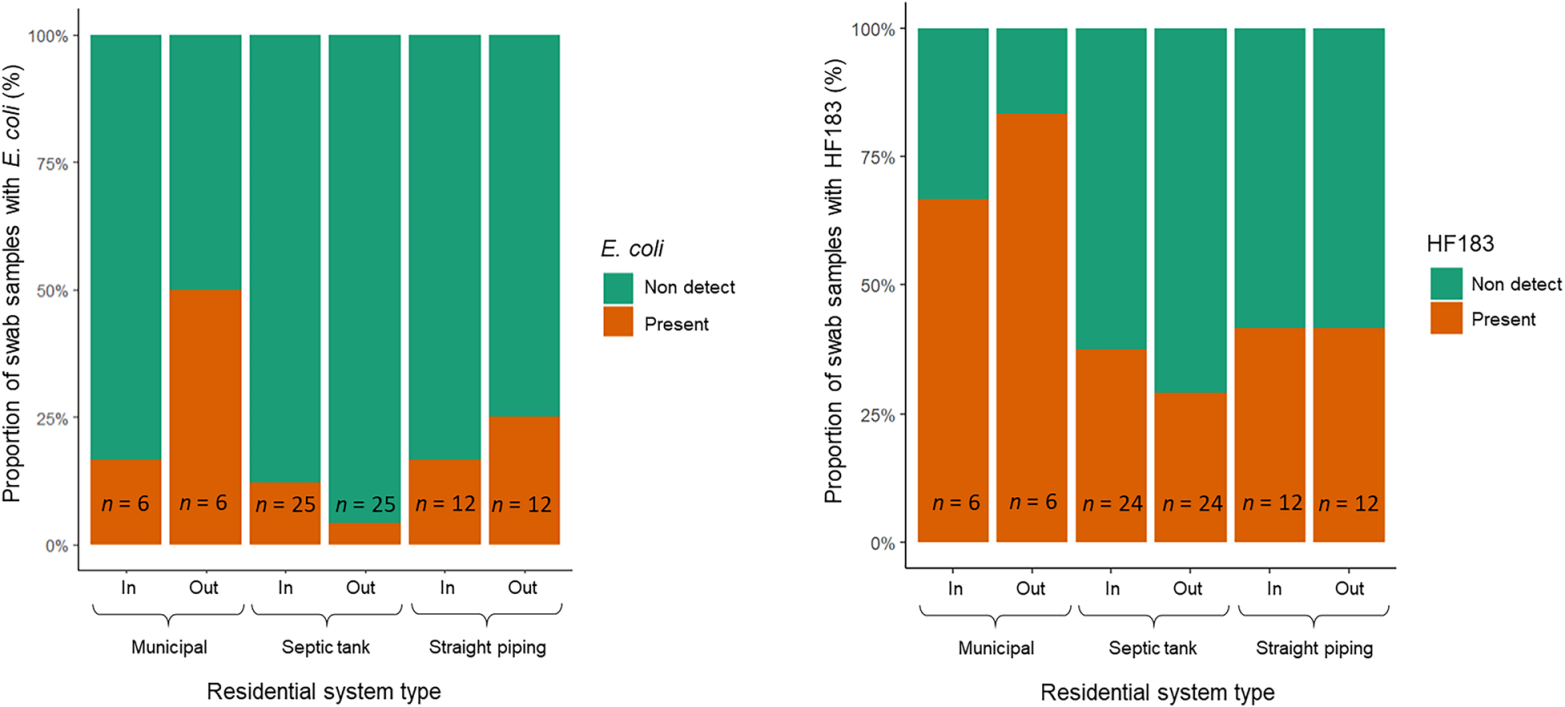
Proportion of houses which had detectable levels of *Escherichia* *coli* or HF183 in residential swab samples for inside and outside doorsteps

**Fig. 7:**
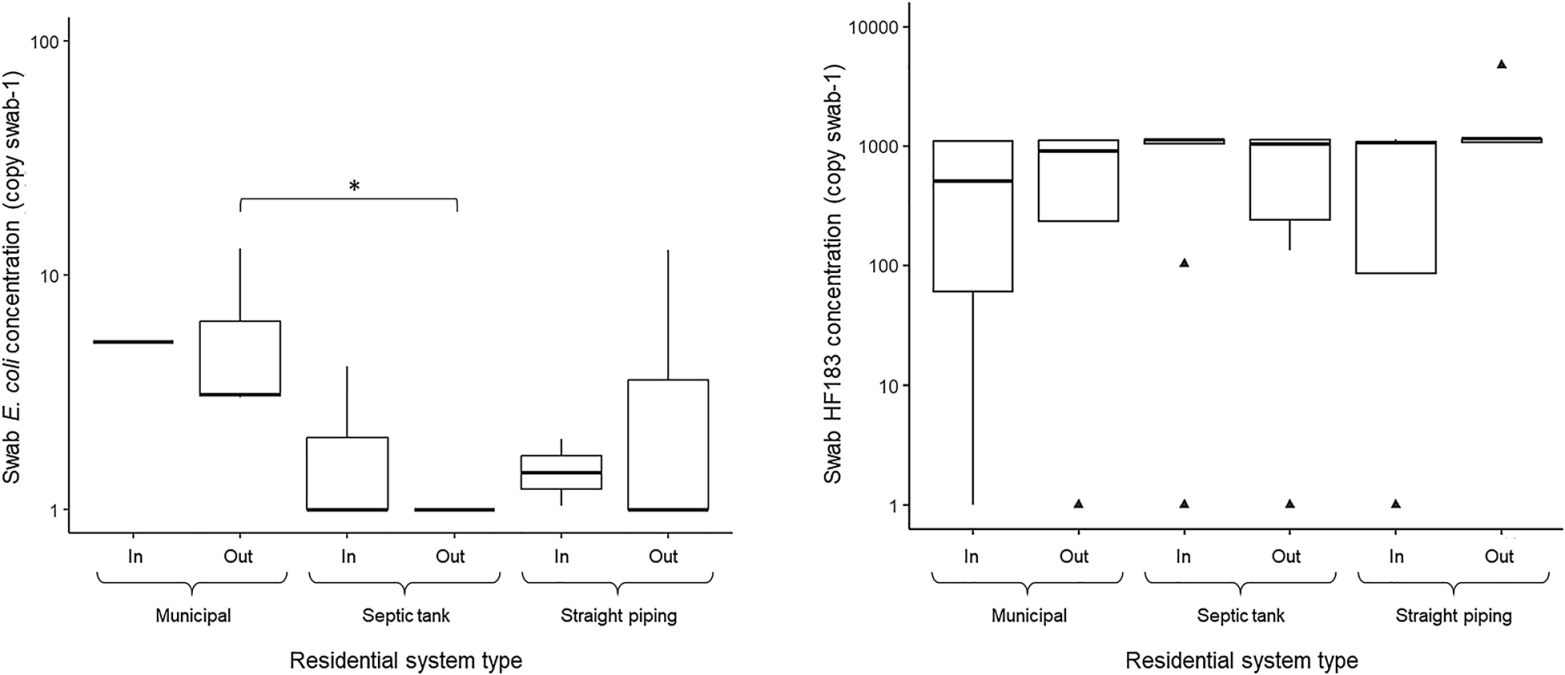
Boxplots of average quantifiable *Escherichia* *coli* or HF183 in residential swab samples for inside and outside doorstep; Samples above the qPCR limit of detection and below the limit of quantification are represented as 1 copy/swab; ^▲^outliers; *statistical significance (*P* < 0.05) from ANOVA test

Of the 43 houses sampled, 30% total tested positive for culturable *E. coli* presence on doorsteps, as shown in Fig. 4. When the residences were broken down by sanitation system type, 50%, 20%, and 42% had detectable amounts of *E. coli*, for municipal, septic tank, and straight piping systems, respectively. Samples ranged from non-detect to 13 *E. coli* CFUs swab^−1^, a few samples reaching the upper limit of detection even with one 1∶10 dilution of IDEXX tests at 4840 CFUs swab^−1^. For the house swab samples which had quantifiable amounts of *E. coli*, concentrations are shown in Figs. 5, 6, 7. A Tukey test determined a statistically significant difference (*P* < 0.05) between the average swab *E. coli* concentration between houses with septic tank and municipal system types. However, within sanitation type groups the difference of *E. coli* concentration was not affected (*P* > 0.05) by a doormat being located inside compared to outside.

There were more houses with *E. coli* CFUs present on their outside doormats than on their inside ones (16% versus 14%). For the HF183 marker, 55% of 42 house swabs yielded detectable amounts of the target gene in their doormats. When compared to inside and outside doormat swabs for the total number of households, 43% of inside and 40% of outside doormat swabs had presence of HF183 gene copies. Eighty three percent, 50%, and 50% of the swab samples that were associated with municipal services, septic tank, and straight piping systems, respectively, had detectable amounts of the HF183 gene marker. Concentrations ranged from non-detect to 4742 gene copies per swab and also demonstrated no statistically significant difference (*P* > 0.05) in the average concentration among sanitation system types, as per Tukey test.

A Spearman non-parametric correlation coefficient analysis determined that there was no statistical association (*P* > 0.05) of the concentration of *E. coli* on the concentration of HF183 genes and vice versa. There was a greater number of samples that had detectable amounts of the HF183 gene than those that had culturable *E. coli*, with residences connected to municipal sewerage having the largest presence of each (Fig. 4). For all sanitation system types, linear regression analysis demonstrated a statistically significant relationship (*P* < 0.05) between concentration of *E. coli* found in doorstep surface swabs with concentration of *B. hominis* found in corresponding residential soil samples.

Logistic regression analysis demonstrated that the presence of *E. coli* or HF183 genes on residential swabs did not correlate (*P* > 0.05) with a presence of *E. coli* or HF183 presence in soil samples from the same residences. This analysis rejects the hypothesis that fecal indicators are transported from residential soil to doormats by adhering and detaching from shoes. However, separate linear regression analysis determined that with respect to houses equipped with straight piping systems, the concentration of HF183 genes measured in soil samples was a significant (*P* < 0.05) predictor of the concentration of *E. coli* in corresponding swab samples. The residences which had a septic tank system had both the fewest proportion of *E. coli* present in soil samples and the fewest proportion of *E. coli* present in doorstep swab samples. Houses connected to municipal systems had the highest percent presence of *E. coli* and human-specific markers in both their soil and swab samples and the highest concentrations of culturable *E. coli* in surface swabs.

### Environmental site selection

During the study period, we collected soil and water samples from 18 different environmental surface water bodies that are publicly accessible (see Table 1). All environmental sampling sites were areas of interest identified by both participating residents and CREEJ that were in close proximity to the participating households that were used for irrigation, fishing, recreation, etc. on a regular basis in all Lowndes County districts, excluding Fort Deposit.

### Fecal markers & parasites in environmental surface water samples

**Fig. 8:**
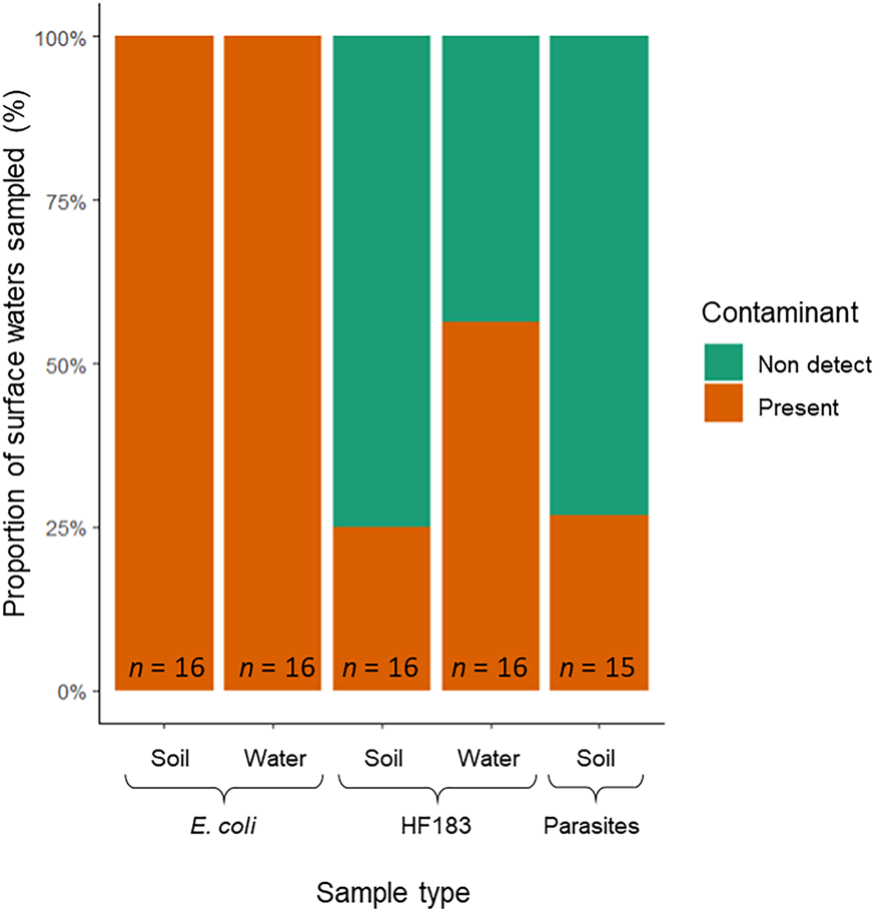
Proportion of surface water samples which had detectable levels of *Escherichia* *coli*, HF183 and parasites for soil and water sub-component samples

**Fig. 9:**
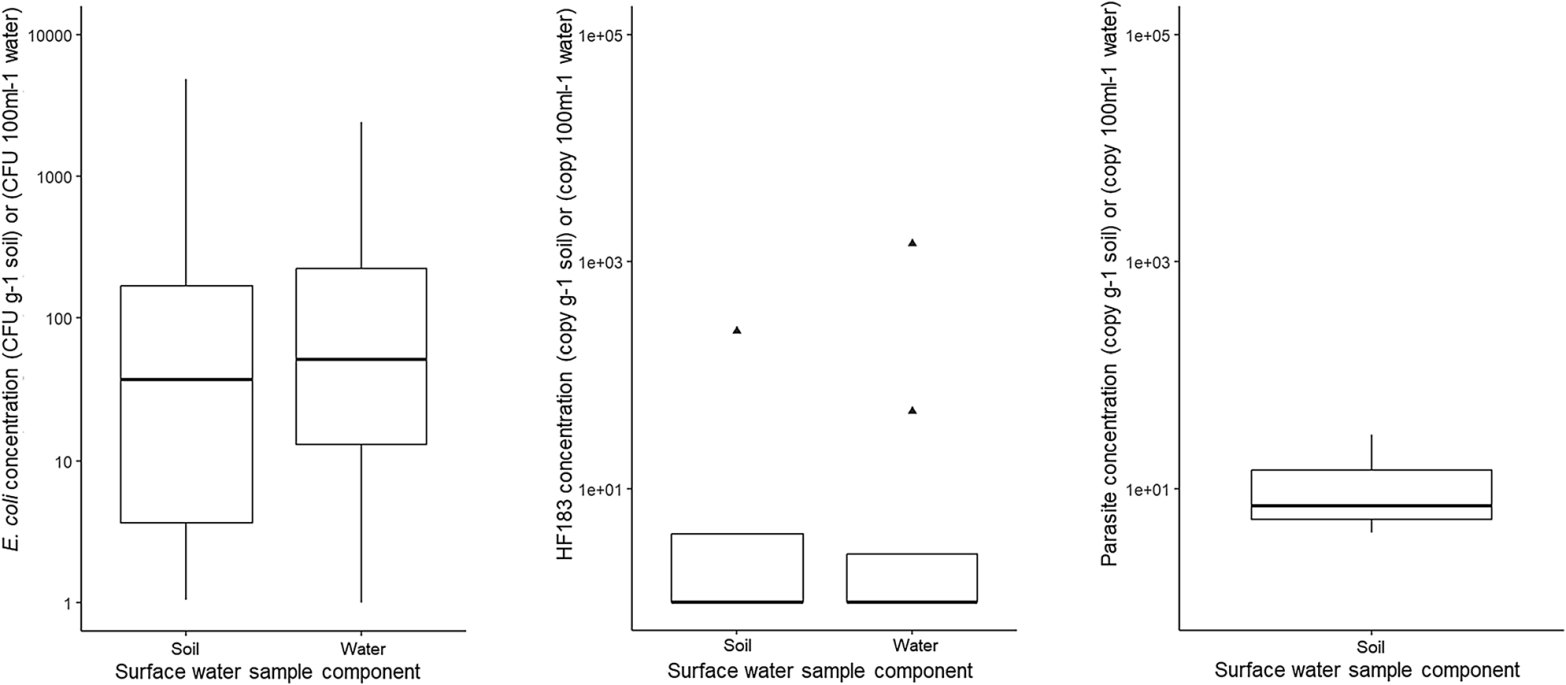
Boxplots of average quantifiable *Escherichia* *coli*, HF183 and parasites concentrations in surface water samples for soil and water sub-component samples. Samples above the qPCR limit of detection and below the limit of quantification are represented as 1 copy/g soil or 1 copy/100 ml water, ^▲^outliers

Of the 18 surface water samples that were sampled, 100% tested positive for *E. coli*, found in both soil and water subsample (Figs. 8, 9). The range of *E. coli* concentration was 1 to 4840 CFU g^−1^ and 1 to 2420 CFU 100 ml^−1^ for the soil subsample and water subsample and, respectively.

There was a higher percent HF183 presence found in the water subsample components per 100 ml compared to the soil components per gram. Of 18 environmental locations, 50% had detectible amounts of the fecal marker. For the soil subsample and water subsample, HF183 copy concentration ranged from non-detect to 2.43 × 10^2^ copies g^−1^ wet soil and from non-detect to 1.44 × 10^3^ copies 100 ml^−1^, respectively.

For the soil component of environmental surface water samples, 7% tested positive for *Entamoeba histolytica* and 20% for *B. hominis*. All samples had non-detect levels for *A. duodenale, A. lumbricoides, N. americanus, Trichuris trichiura, S. stercoralis, G. intestinalis, Cryptosporidium, Toxocara canis, Toxocara cati*. Of the environmental samples, four locations (27%) tested positive for anthropogenic parasites. Of the 15 environmental samples that were tested for *E. coli*, HF183, and parasites, 3 of the locations (20%) had soil samples that tested positive for all three categories simultaneously.

## Discussion

### Fecal markers in residential drinking water & soil

There was not a presence of detectable culturable *E. coli* in all of the 43 residential drinking water samples. This indicates that for the houses sampled, there was no fecal contamination of their drinking water regardless of corresponding sanitation infrastructure that is present at the house.

Of those tested, 88% of all residences tested positive for *E. coli* in soil samples. This proportion is higher than a study conducted in rural households in Bangladesh where improved sanitation coverage was 29%, and 42% of residential soil samples tested positive for *E. coli* with an average concentration of 89 CFUs g^−1^ dry soil [18].

*E. coli* was present in residential soils at a higher rate than presence of HF183 human fecal markers for each sanitation system group type. This phenomenon might be partially explained by work highlighting temperature and water availability as two of the most significant factors which influence the presence of *E. coli* in soil [20]. Most of Lowndes County has soil with high clay content through which rain and wastewater does not percolate well [11]. This allows the soil to hold water and as a result can serve as rehydration for desiccated organisms and can create a favorable anoxic environment for *E. coli* and other anaerobes to thrive [21].

The HF183 concentrations found in sampled residential soils is comparable to the mean HF183 concentration of 1.73 × 10^4^ copies g^−1^ measured in soil samples immediately surrounding latrines in Mozambique [22]. However, the percent of samples with HF183 presence in residential soil in this study was about 25% more than found by Holcomb et al. (38%) and over three times the proportion found in soil samples of Tanzanian residences without improved sanitation [23]. As *E. coli* presence in the soil demonstrate microbial survival and the HF183 gene has a half-life (the time it takes for half of the DNA matter to decay) of 6–8 days the presence of both culturable *E. coli* and HF183 demonstrate the proliferation of fresh fecal contamination from human sources in Lowndes residential soil [24, 25].

### Fecal markers in residential swabs

There were more houses with *E. coli* CFUs present on their outside doormats than on their inside ones (16% versus 14%). This is interesting, as outdoor mats having more exposure to UV which can inactivate *E. coli* and might expect less prevalence [20]*.* The variability of floor surface types could also affect *E. coli* recovery for swab applications [26]. Porous surfaces could be more conducive to bacteria survival and could yield higher capture rates. Though on the contrary, collecting from porous surfaces might not capture as much bacteria, as microbes could be shielded within the pores [26]. Since surface type for each swab sample was not recorded, it is difficult to speculate if doormat surface type was a significant factor of *E. coli* detection rates in swab samples.

HF183 markers were present in residential doorstep swabs at rates of 83, 5, and 50% for houses that that municipal, septic tank, and straight piping infrastructure, respectively. This is similar to a study that found over 70% of houses in the Kibera urban slum in Nairobi, Kenya had floor swabs which tested positive for both culturable *E. coli* and HF183 genes [27]. The location of a doormat, inside or outside, did not correlate with a significant difference in concentration, for each sanitation system type. This trend for both *E. coli* and HF183 concentration suggests that swab concentrations vary with respect to sanitation system type but the concentration of indicators on the indoor and outdoor doormats did not vary within the same sanitation system group.

There was a greater number of residential door step swab samples that had detectable amounts of the HF183 gene than that had culturable *E. coli*, with residences on municipal systems having the largest presence of each. This is consistent with a study showing that indicator organism presence using molecular biology methods was twice as much as when using culture-based methods [22]. This could be due to HF183 being a DNA gene which could be present in a variety of organisms compared to detecting the presence of viable *E. coli* CFUs. As doormats are less favorable environments for microbes than soil, *E. coli* and other microorganisms could have been transported from residential soils to doormats where they would have lysed due to lack of nutrients, exposure to UV, etc. but the human-specific markers were still able to be captured and detected. Another possible reason that might explain why there was a greater presence of HF183 than *E. coli* in swab samples versus soils samples is that soils are complex environmental samples which are known for containing PCR inhibitors [28]. Whether or not residents typically walk on an *E. coli* positive section of soil before entering their residence could be a factor in *E. coli* presence or concentration in doormat swab samples. If *E. coli* is present in a resident’s soil, the proximity of the *E. coli* positive soil to the door of entry could also be a possible significant factor. If fecal bacteria are being transported by shoes into the residence, and if people customarily wipe their feet on the area outside the door before entering, a higher presence of fecal bacteria could be expected in the outside swab samples than the inside swab samples.

Houses connected to municipal systems had the highest percent presence of *E. coli* and human-specific markers in both their soil and swab samples and the highest concentrations of culturable *E. coli* in surface swabs. These findings suggest that the presence of *E. coli* and HF183 genes in the soil could be related to their presence on doormats of residents, regardless of what type of sanitation system a residence has. Reports from residents who have experienced sanitation system failures suggest that raw sewage exposure was related to the clayey soil which comprised most of Lowndes County [29]. This context aligns with our findings.

We originally assumed that the residences connected to municipal systems would have less fecal exposure than residences which have septic tank or straight pipe systems, as wastewater should theoretically be confined to pipes and transported away. However, residents connected to municipal systems have reported sewage not only flooding their yard but also backing up into their homes through the toilet, showers, and the sinks. This context could very well be a significant factor to explain our findings although the participants were not surveyed for incidences of sewage backing up as a part of the study.

### Fecal markers in environmental surface water samples

It is recommended by the EPA not to come in contact with water when *E. coli* concentrations exceed 235 CFUs per 100 ml [30]. Of the 18 surface water samples collected, there were three samples, two streams and one pond, that had culturable *E. coli* concentrations exceeding the EPA recommend limit for contact. These sample locations should not be used for recreation, irrigation, or any other anthropogenic purposes, but were identified by CREEJ and participating residents that they are commonly used for such purposes.

Although the concentration of culturable *E. coli* and HF183 marker is less than a study reporting 80% of samples from standing water, drainage ditches, and streams testing positive for both culturable *E. coli* and HF193 genes [27], the fact that half of the samples were positive for *E. coli* and HF183 demonstrates the severity of fecal matter contamination. One surface water sample exceeded the HF183 risk-based water quality threshold of 525 copies 100 ml^−1^ [31] with 1440 copies 100 ml^−1^. This demonstrates heavy contamination of surface waters with fecal material.

Of the 15 environmental samples that were tested for *E. coli*, HF183, and parasites, 3 of the locations (20%) had soil samples that tested positive for all three categories simultaneously. Similar to studies in the built environment, the presence of parasites was detected alongside the other markers of fecal contamination.

### Parasites in residential and environmental soil

There is a well-known connection between socioeconomic status and the risk of parasitic infections. Doni et al. highlighted that poor socioeconomic conditions, combined with children’s behavior of playing in soil, significantly increased the risk of parasitic infections in a cohort of Turkish children [32]. Overall, our study highlights the outcome of inadequate sanitation infrastructure. In many rural areas of the United States, residents often lack access to municipal sanitation services and instead depend on septic tanks, which can overflow or malfunction without proper maintenance, leading to a higher risk of exposure to raw sewage and, consequently, an increased risk of parasitic infections.

Of the 38 residences that were tested for *E. coli*, HF183, and parasites, 10 of the houses (26%) had soil samples that tested positive for all three categories simultaneously, indicating potential for exposure to harmful bacteria and parasites of fecal origin. These results add further evidence that human fecal contamination is common surrounding the built environments in Alabama. The greatest incidence of parasites in our soil samples was for *B. homini*s, a gastrointestinal protozoa among the most commonly reported in tropical and subtropical countries [33]. Detection rates of other parasites were low. A single sample tested positive for *A. lumbricoides*, and none were positive for *N. americanus* (i.e., hookworm), *A. duodenale, S. stercoralis, Cryptosporidium* species*,* and *G. intestinalis*, in relatively good agreement (except for *Giardia*) with an earlier study using similar sampling methodology conducted in the Paiute tribal band lands in Utah [34]. The lower incidence rate of parasites in the soil samples, compared to the more prevalent detection of fecal indicators, suggests that while contamination with fecal material is evident, the occurrence, survival and/or transmission of parasites could be more limited under the environmental conditions observed.

## Conclusions

Environmental monitoring of fecal indicators in Lowndes County using culture-based and molecular biology-based methods suggest that while residential drinking water samples did not have presence of fecal bacteria or fecal indicators, residential soils and residential doorsteps have widespread presence of human fecal indicator organisms or genes, regardless of what type of sanitation system the residence has. Human and zoonotic parasites were present in residential soils of all different sanitation system types, but at much lower rates compared to fecal indicators. In addition to residential properties, the proliferation of human fecal contaminants is spread throughout the environment and impacts bodies of surface water to levels which exceed Federal limits. This study provides evidence that failing sanitation infrastructure is prevalent in all districts sampled in Lowndes County. Our findings also challenge a common narrative that exposure to untreated human waste would be eradicated if municipal systems were implemented. Our findings support the narrative that sanitation infrastructure of all types in Lowndes County, Alabama is compromised and highlights both residential and environmental environments are subjected to raw wastewater exposure and a variety of associated biological hazards. Current infrastructure efforts such as the Bipartisan Infrastructure Law, Justice 40, the Inflation Reduction Act, and others need to address the failures of all sanitation system types in Lowndes County.

## Data availability

All data generated or analyzed during this study are included in this published article and its supplementary information files.

## Supplementary Information

Supplementary Material 1.

## Data Availability

All data analyzed during this study are included in this published article and its supplementary information files.

## Abbreviations

CFU: Colony forming units
DI: Deionized
IRB: Institutional Review Board
LOD: Limit of detection
LOQ: Limit of quantification
MPN: Most probable number
qPCR: Quantitative polymerase chain reaction
UV: Ultraviolet

## References

[1] World Health Organization, United Nations Children’s Fund. Progress on household drinking water, sanitation and hygiene 2000–2020. Geneva: World Health Organization; 2021.

[2] ASCE. Failure to act: the economic impact of current investment trends in water and wastewater infrastructure. Virginia: ASCE; 2011.

[3] U.S. EPA. Septic systems overview. Washington, DC: U.S. EPA.; 2015.

[4] Gasteyer SP, Lai J, Tucker B, Carrera J, Moss J. BASICS INEQUALITY: race and access to complete plumbing facilities in the United States. Bois Rev Soc Sci Res Race. 2016;13(2):305–25. 10.1017/S1742058X16000242.

[5] Gasteyer SP, Vaswani R. Without the basics analyzing the availability of water and sanitation services in the United States rural community assistance partnership in the 21st century still living. New York: Polity Press; 2004. p. 1–208.

[6] ASCE. 2017 Infrastructure report card: wastewater. 2017. https://www.infrastructurereportcard.org/wp-content/uploads/2017/01/Wastewater-Final.pdf.

[7] ASCE. Closing the infrastructure for america’s economic future investment gap to failure to act: the impact of infrastructure. Virginia: ASCE; 2020.

[8] de Albuquerque. Report of the special rapporteur on the human right to safe drinking water and sanitation. Mission to the United States of America. 2011. 1–21.

[9] United Church of Christ. Toxic wastes and race in the United States: A national report on the racial and socio-economic characteristics of communities with hazardous waste sites. 1987.

[10] US Water Alliance. Closing the water access gap in the United States. 2019.

[11] He J, Dougherty M, Zellmer R, Martin G. Assessing the status of onsite wastewater treatment systems in the Alabama black belt soil area. Environ Eng Sci. 2011;28(10):693–9. 10.1089/ees.2011.0047.

[12] McKenna ML, McAtee S, Bryan PE, Jeun R, Ward T, Kraus J, et al. Human intestinal parasite burden and poor sanitation in rural Alabama. Am J Trop Med Hyg. 2017;97(5):1623–8. 10.4269/ajtmh.17-0396. 29016326 10.4269/ajtmh.17-0396 PMC5817782

[13] Poole C, Barker T, Bradbury R, Capone D, Chatham AH, Handali S, et al. Cross-sectional study of soil-transmitted helminthiases in black belt region of Alabama, USA. Emerg Infect Dis. 2023. 10.3201/eid2912.230751. 37987581 10.3201/eid2912.230751 PMC10683802

[14] Sherpa AM, Koottatep T, Zurbrügg C, Cissé G. Vulnerability and adaptability of sanitation systems to climate change. J Water Clim Change. 2014;5(4):487–95. 10.2166/wcc.2014.003.

[15] Alderman K, Turner LR, Tong S. Floods and human health: a systematic review. Environ Int. 2012;47:37–47. 10.1016/j.envint.2012.06.003. 22750033 10.1016/j.envint.2012.06.003

[16] Howard G, Calow R, Macdonald A, Bartram J. Climate change and water and sanitation: likely impacts and emerging trends for action. Annu Rev Environ Resour. 2016;41(1):253–76. 10.1146/annurev-environ-110615-085856.

[17] Mejia R, Seco-Hidalgo V, Garcia-Ramon D, Calderón E, Lopez A, Cooper PJ. Detection of enteric parasite DNA in household and bed dust samples: potential for infection transmission. Parasit Vectors. 2020. 10.1186/s13071-020-04012-6. 32188497 10.1186/s13071-020-04012-6 PMC7079405

[18] Montealegre MC, Roy S, Böni F, Hossain MI, Navab-Daneshmand T, Caduff L, et al. Risk factors for detection, survival, and growth of antibiotic-resistant and pathogenic *Escherichia coli* in household soils in rural Bangladesh. Appl Environ Microbiol. 2018;84(24):e01978-e2018. 10.1128/AEM.01978-18. 30315075 10.1128/AEM.01978-18 PMC6275341

[19] Green HC, Haugland RA, Varma M, Millen HT, Borchardt MA, Field, et al. Improved HF183 quantitative real-time PCR assay for characterization of human fecal pollution in ambient surface water samples. Appl Environ Microbiol. 2014;80(10):3086–94. 10.1128/AEM.04137-13. 24610857 10.1128/AEM.04137-13 PMC4018914

[20] Jang J, Hur H-G, Sadowsky MJ, Byappanahalli MN, Yan T, Ishii S. Environmental *Escherichia coli*: ecology and public health implications-a review. J Appl Microbiol. 2017;123(3):570–81. 10.1111/jam.13468. 28383815 10.1111/jam.13468

[21] Van Elsas JD, Semenov AV, Costa R, Trevors JT. Survival of *Escherichia coli* in the environment: fundamental and public health aspects. ISME J. 2011;5(2):173–83. 10.1038/ismej.2010.80. 20574458 10.1038/ismej.2010.80 PMC3105702

[22] Holcomb DA, Knee J, Sumner T, Adriano Z, De Bruijn E, Nalá R, et al. Human fecal contamination of water, soil, and surfaces in households sharing poor-quality sanitation facilities in Maputo, Mozambique. Int J Hyg Environ Health. 2020;226: 113496. 10.1016/j.ijheh.2020.113496. 32135507 10.1016/j.ijheh.2020.113496 PMC7174141

[23] Pickering AJ, Julian TR, Marks SJ, Mattioli MC, Boehm AB, Schwab KJ, et al. Fecal contamination and diarrheal pathogens on surfaces and in soils among Tanzanian households with and without improved sanitation. Environ Sci Technol. 2012;46(11):5736–43. 10.1021/es300022c. 22545817 10.1021/es300022c

[24] Seurinck S, Defoirdt T, Verstraete W, Siciliano SD. Detection and quantification of the human-specific HF183 Bacteroides 16S rRNA genetic marker with real-time PCR for assessment of human faecal pollution in freshwater. Environ Microbiol. 2005;7:249–59. 15658992 10.1111/j.1462-2920.2004.00702.x

[25] Walters SP, Field KG. Survival and persistence of human and ruminant-specific faecal Bacteroidales in freshwater microcosms. Environ Microbiol. 2009;11:1410–21. 19397677 10.1111/j.1462-2920.2009.01868.x

[26] Valentine NB, Butcher MG, Su Y-F, Jarman KH, Matzke M, Webb-Robertson B-J, et al. Evaluation of sampling tools for environmental sampling of bacterial endospores from porous and nonporous surfaces. J Appl Microbiol. 2008;105(4):1107–13. 10.1111/j.1365-2672.2008.03840.x. 18492049 10.1111/j.1365-2672.2008.03840.x

[27] Bauza, V. (2017). Elucidating Fecal Contamination Exposure In Low-Income Countries, The Contribution From Child Feces Disposal Practices And Soil Ingestion, And Links To Child Health.

[28] Schrader C, Schielke A, Ellerbroek L, Johne R. PCR inhibitors—occurrence, properties and removal. J Appl Microbiol. 2012;113:1014–26. 22747964 10.1111/j.1365-2672.2012.05384.x

[29] Albright EA, Coleman Flowers C, Cramer RA, Weinthal ES. Failing septic systems in Lowndes county, Alabama: citizen participation, science, and community knowledge. Local Environ. 2023. 10.1080/13549839.2023.2267066.

[30] U.S. EPA. Ambient water quality criteria for bacteria. 1986

[31] Boehm AB, Soller JA. Refined ambient water quality thresholds for human-associated fecal indicator HF183 for recreational waters with and without co-occurring gull fecal contamination. Microb Risk Analy. 2020. 10.1016/j.mran.2020.100139.

[32] Yentur Doni N, Yildiz Zeyrek F, Simsek Z, Gurses G, Sahin I. Risk factors and relationship between intestinal parasites and the growth retardation and psychomotor development delays of children in Şanlıurfa, Turkey. Turk J Parasitol. 2016;39(4):270–6. 10.5152/tpd.2015.3620. 10.5152/tpd.2015.3620 26809913

[33] Ocaña-Losada C, Cuenca-Gómez JA, Cabezas-Fernández MT, Vázquez-Villegas J, Soriano-Pérez MJ, Cabeza-Barrera I, et al. Clinical and epidemiological characteristics of intestinal parasite infection by Blastocystis hominis. Rev Clínica Esp. 2018. 10.1016/j.rce.2018.01.003. 10.1016/j.rce.2018.01.003 29455921

[34] McKim S, Kopystynsky K, Wolf N, Akbar F, Bottazzi ME, Hotez PJ, et al. Environmental detection of parasites in the marginalized Paiute reservations compared to a nearby area. Am J Trop Med Hyg. 2023. 10.1101/2023.10.24.23297407. 10.4269/ajtmh.23-0712 PMC10919181 38350146

