# Supplementary material for: Impact of sanitation system types on residential and environmental presence of human waste and parasites in Alabama
Source: Infect Dis Poverty. 2025 Jul 11;14:65. doi: 10.1186/s40249-025-01334-4 (PMC12247418; doi:10.1186/s40249-025-01334-4)
Supplement: Supplementary file 1 — Supplementary Material 1. [file 40249_2025_1334_MOESM1_ESM.docx]

Supplemental section

Supplemental Table 1. Environmental Microbiology Minimum Information (EMMI) for qPCR.

| **Environmental Sampling** | **qPCR** |
| --- | --- |
| Up to 50 grams | Target genes (supplemental table 2) |
| Alabama, USA | Hold stage 95°C, 20 sec; Amplification Denaturation 95°C, 1 sec; Annealing 60°C, 20 sec. |
| July 2019 | 2x TaqMan® Fast Advanced Master Mix (Applied Biosystems, Foster City, CA) 3.5 µl |
| Stored at 4°C and DNA extracted within 1 month | 2 µl of template |
| Exogenous DNA was used as an internal control to validate the extraction method. All samples had the internal control detect via qPCR | Primers were used at 900 nM (Thermofisher)  Probe was used at 100 nM (Thermofisher) |
| **Sample Treatment** | ViiA 7 Fast Real-time PCR System (Applied Biosystems, Waltham, Massachusetts, USA) |
| Soil washed with PBS and 0.05% Tween 20 | 2 µl of PCR-water was used as a negative control |
| Flotation with 35.6% Sodium Nitrate solution | Plasmids containing target parasite gene sequences was used as positive control |
| **Sample Reduction** | An exogenous DNA internal control was tested and all samples tested positive for the internal control Ct median  33.675. |
| Samples are concentrated by a factor of 500 | **Analysis - qPCR** |
| **Nucleic Acid Extraction** | Positive control standard curves were performed in duplicate |
| MP fastDNA Spin kits for soil | Samples were tested in single |
| DNA eluent is 100 µl and stored at -20°C | All positive controls were compared to a set of known Ct values and were all within 5% range |
|  | Lowest standard measured was approximately 10 fg |
|  | Automatic baseline and a threshold of 0.04 was used for all parasites |

Supplemental Table 2.

**TABLE 2.** Target regions, primer sequences, and probe sequences by parasites for DNA amplification.*

| Parasite | Target region | Forward primer sequence (5’ to 3’)  Reverse primer sequence (5’ to 3’)  Probe sequence (5’FAM to 3’) |
| --- | --- | --- |
| *Ancylostoma duodenale* | ITS-2 | GAATGACAGCAAACTCGTTGTTG ATACTAGCCACTGCCGAAACGT ATCGTTTACCGACTTTAG |
| *Ascaris lumbricoides* | ITS-1 | TGCACATAAGTACTATTTGCGCGTAT  CCGCCGACTGCTATTACATCA  GAGCCACATAGTAAATT |
| *Cryptosporidium* spp*.* | DNA-J like protein | AACTTCACGTGTGTTTGCCAAT  CCAATCACAGAATCATCAGAATCG  CATATGAAGTTATAGGGATACCAG |
| *Blastocystis* spp*.* | 16s rRNA | AGTAGTCATACGCTCGTCTCAAA  TCTTCGTTACCCGTTACTGC  CGTGTAAATCTTACCATTTAGAGGA |
| *Entamoeba histolytica* | 18S rRNA | GTTTGTATTAGTACAAAATGGCCAATTC  TCGTGGCATCCTAACTCACTTAGA  CAATGAATTGAGAAATGACA |
| *Giardia intestinalis* | 16S rRNA | CATGCATGCCCGCTCA  AGCGGTGTCCGGCTAGC  AGGACAACGGTTGCAC |
| *Necator americanus* | ITS-2 | CTGTTTGTCGAACGGTACTTGC  ATAACAGCGTGCACATGTTGC  CTGTACTACGCATTGTATAC |
| *Strongyloides stercoralis* | 18s rRNA | GAATTCCAAGTAAACGTAAGTCATTAGC  TGCCTCTGGATATTGCTCAGTTC  ACACACCGGCCGTCGCTGC |
| *Toxocara canis* | ITS-2 | GCGCCAATTTATGGAATGTGAT  GAGCAAACGACAGCSATTTCTT  CCATTACCACACCAGCATAGCTCACCGA |
| *Toxocara cati* | ITS-2 | ACGCGTACGTATGGAATGTGCT  GAGCAAACGACAGCSATTTCTT  TCTTTCGCAACGTGCATTCGGTGA |
| *Trichuris trichiura* | ITS-1 | TCCGAACGGCGGATCA  CTCGAGTGTCACGTCGTCCTT  TTGGCTCGTAGGTCGTT |
| *Internal Amplification Control (IAC)* | Synthetic sequence | CTAACCTTCGTGATGAGCAATCG  GATCAGCTACGTGAGGTCCTAC  TCGATGCACTCCAGTCCTCCT |
| *ITS = internal transcribed spacer; rRNA = ribosomal RNA | |  |

Supplemental Table 3: Average quantities of residential swab and soil samples

Supplemental Table 4: Average quantities of environmental water and soil samples
